# Urinary l-*erythro*-β-hydroxyasparagine—a novel serine racemase inhibitor and substrate of the Zn^2+^-dependent d-serine dehydratase

**DOI:** 10.1042/BSR20210260

**Published:** 2021-04-23

**Authors:** Tomokazu Ito, Mayuka Tono, Yasuyuki Kitaura, Hisashi Hemmi, Tohru Yoshimura

**Affiliations:** Department of Applied Biosciences, Graduate School of Bioagricultural Sciences, Nagoya University, Furou-chou, Chikusa, Nagoya, Aichi 464-8601, Japan

**Keywords:** amino acids, D-serine, hydroxyasparagine, pyridoxal phosphate, serine racemase

## Abstract

In the present study, we identified l-*erythro*-β-hydroxyasparagine (l-β-EHAsn) found abundantly in human urine, as a novel substrate of Zn^2+^-dependent d-serine dehydratase (DSD). l-β-EHAsn is an atypical amino acid present in large amounts in urine but rarely detected in serum or most organs/tissues examined. Quantitative analyses of urinary l-β-EHAsn in young healthy volunteers revealed significant correlation between urinary l-β-EHAsn concentration and creatinine level. Further, for in-depth analyses of l-β-EHAsn, we developed a simple three-step synthetic method using *trans*-epoxysuccinic acid as the starting substance. In addition, our research revealed a strong inhibitory effect of l-β-EHAsn on mammalian serine racemase, responsible for producing d-serine, a co-agonist of the *N*-methyl-d-aspartate (NMDA) receptor involved in glutamatergic neurotransmission.

Living organisms use 20 different amino acids to form various proteins. Although a large number of amino acids present in biological fluids are proteinogenic l-amino acids, a substantial amount of non-proteinogenic l- and d-amino acids have also been detected and are known to possess several physiological functions [1–4]. One such example is the mammalian d-Ser. This amino acid comprises one-fourth of the total serine present in the central nervous system and plays an important role in memory and learning by modulating the glutamatergic *N*-methyl-d-aspartate (NMDA) receptor [5]. d-Ser is endogenously produced from l-Ser by the pyridoxal 5′-phosphate-dependent enzyme, serine racemase (SR) [6,7]. d-amino acid oxidase (DAO), an FAD-containing flavoenzyme is responsible for degrading d-Ser in mammals [8–10]. Recent studies have suggested the implication of changes in d-Ser levels in various NMDA receptor-linked neurological disorders, including schizophrenia, epilepsy, amyotrophic lateral sclerosis, and kidney injury [11–14].

In several non-mammalian eukaryotic organisms, an alternate d-Ser-degrading enzyme, namely d-serine dehydratase (DSD), is present. DSD specifically degrades d-Ser in a Zn^2+^- and PLP-dependent manner [15]. In chickens, DSD is localized in the proximal tubule cells of the kidney, hepatocytes, Bergmann-glia cells of the cerebellum, and astrocytes and is involved in NMDA receptor-dependent brain functions [16,17]. In *Dictyostelium discoidem*, a cellular slime mold, DSD, but not DAO, is preliminarily responsible for the decomposition of d-Ser. In addition, a *dsd*-deficient *D. discoideum* strain showed accumulation of d-Ser and delayed development; the latter was found to be caused by perturbations in the cAMP signaling relay [18]. DSD isolated from *Saccharomyces cerevisiae* (Dsd1p) displayed high substrate specificity for d-Ser, while l-Ser was inert as a substrate [19]. Recent studies have suggested the possibility of evaluating certain neurological and/or renal disorders by analyzing d-Ser levels in serum and urine [20,21]. Accordingly, we developed a novel enzymatic d-/l-Ser assay system that uses Dsd1p and lactate dehydrogenase or pyruvate oxidase for high-throughput determination of d- and/or l-Ser concentration in serum and urine [19,22].

Our prior investigation associated with the study of urinary d-Ser revealed the presence of a previously unknown amino acid in human urine that was degraded by Dsd1p. Thus, the present study was aimed to identify this novel amino acid. We identified the unknown amino acid as l-*erythro*-β-hydroxyasparagine (l-β-EHAsn). Furthermore, kinetic studies related to urinary l-β-EHAsn excretion in healthy young volunteers revealed a strong correlation between urinary l-β-EHAsn concentration and creatinine level. In addition, we describe a novel simple three-step method for the synthesis of l-β-EHAsn and show the strong inhibitory effect of l-β-EHAsn on mammalian SR.

## Materials and methods

### Materials

l-*erythro*-β-hydroxyaspartate was purchased from Wako-Fujifilm (Osaka, Japan). *Trans*-epoxysuccinic acid was obtained from Tokyo Kasei (Tokyo, Japan). dl-*threo*-β-hydroxyaspartic acid and d-*threo*-β-hydroxyaspartic acid were a gift from Dr. Masaru Wada from Hokkaido University.

### Urine samples

The spot urine samples (collected at first-morning and at random at noon and night for 3 days) were provided by 20 young healthy volunteers (male/female: 10/10, age range: 21−25 years). Each individual provided written informed consent for the study. The urinary amino acid analysis was approved by the Committee of the Graduate School of Bioagricultural Sciences, Nagoya University, and carried out under relevant guidelines and regulations to take place.

### Experimental animals

Animal experiments in the present study were approved by the Animal Care Committee of the Graduate School of Bioagricultural Sciences, Nagoya University (Approval No. 2018070501), and were performed at Nagoya University under relevant guidelines and regulations. Six male Sprague–Dawley rats aged 7 weeks were obtained from Japan SLC (Hamamatsu, Japan) and individually housed in wire-mesh cages in a conventional animal room, under the conditions of controlled temperature (23 ± 1°C) and 12-h light–dark cycle (lights on at 8 a.m.). Diets (D12450J, Research Diets, New Brunswick, NJ, U.S.A.) and tap water were supplied *ad libitum*. The 30-week-old rats were fasted for 8 h before sampling. After the deep anesthesia with isoflurane, rats were killed. The liver, kidney, cerebellum, cerebrum, and testes were removed, weighed, and frozen at −80°C until analysis.

### Amino acid analyses

Amino acids in the urine or organs were analyzed using a high-performance liquid chromatography (HPLC) system (Shimadzu, Japan) as previously described [23,24]. Samples were deproteinized with 5% trichloroacetic acid (TCA) as described previously [23]. The amino acids were derivatized with *o*-phthalaldehyde and *N*-acetyl-l-cysteine, separated with an ODS column (Mightysil RP-18 GP-II 150-4.6, 5 μm) (Kanto Chemical, Tokyo, Japan). Excitation and emission wavelengths were 350 nm and 450 nm, respectively.

### Enzyme purification

DSD from *S. cerevisiae* was overexpressed as an N-terminal His-tagged protein in *E. coli* KRX strain (Promega) harboring pET-15b-Dsd1p and purified homogeneity as described previously [15]. SR from mouse (N-terminal His-tag) was overexpressed in *E. coli* Rosetta2 (DE3) harboring pET-28a-mSR and purified as previously described [25]. *E. coli* asparaginase (AnsA) or asparagine synthase (AsnA) was overexpressed as an N-terminal His-tagged protein with strain from the ASKA collection (National BioResource Project (NIG,Japan): *E.coli*) [26]. Briefly, an LB medium (10 g/l polypeptone, 5 g/l yeast extract, 5 g/l NaCl) containing appropriate antibiotics (ampicillin: 100 mg/l, chloramphenicol: 30 mg/l) was used for the cultivation of these *E. coli* strains. IPTG (500 μM) or l-rhamnose (0.02%) was used for the induction of the protein expression. Cells were pelleted by centrifugation and resuspended in a binding buffer (20 mM Tris-HCl, 500 mM NaCl, 20 mM imidazole, pH 7.9). Cells were disrupted by sonication and clarified by centrifugation (20000×***g***, 4°C, 30 min). Cell lysates were applied to Ni^2+^-charged affinity columns (GE Healthcare) and washed with a washing buffer (20 mM Tris-HCl, 500 mM NaCl, 80 mM imidazole, pH 7.9). The target protein was eluted with an elution buffer (20 mM Tris-HCl, 500 mM NaCl, 500 mM imidazole, pH 7.9), and the buffer of the selected fractions was exchanged by PD-10 desalting column (GE Healthcare). The target protein was stored with 10% (v/v) glycerol at −80°C until used.

### LC-ESI-MS analysis

6-Aminoquinoline *N*-succinimidyl ester (AQC) was synthesized as described previously [27]. The partially purified urine sample was incubated with or without Dsd1p (45 μg) in a 50 mM Hepes-NaOH buffer (1.5 ml, pH 8) for 16 h at 30°C. The reaction was terminated by adding TCA (5%). TCA was removed from the sample by three-times extraction with diethyl ether. Seventy microliters of the portion was mixed with 80 μl of 0.4 M borate buffer pH 9.5 and 60 μl of AQC solution (3 mg/ml in dry acetonitrile) and incubated at 55°C for 10 min. After centrifugation (15 min at 20000×***g***), a 100 μl amount of Buffer A (0.14 M ammonium acetate, pH 5.05) was added to the solution. The AQC derivatives were separated by HPLC with a C18 column (Kinetex XB C18, 4.6 × 250 mm, 5 μm) (Phenomenex) and two buffers, Buffer A and Buffer B (acetonitrile/water = 60/40, v/v), as described previously [24]. The eluate was collected every 1 min and analyzed by direct infusion ESI-MS in a positive mode with an Esquire 3000 ion trap system (Bruker Daltonics).

### Preparation of l-β*-*EHAsn

Trans-epoxysuccinic acid (0.26 g) was treated with aqueous concentrated ammonia (25–28%, 7 ml) and incubated at 50°C for 24 h [28]. The solvent was dried under reduced pressure. The residue (l-β-EHAsp and d- β-EHAsp) was dissolved in water. It was further dissolved in a buffer containing 100 mM Hepes-NaOH Buffer (pH 8.0), 20 µM PLP, 1 mM MgCl_2_, 1 mM ATP at a final concentration of 25 mM and incubated with 0.9 mg mouse SR at 37°C for 24 h (final vol. 5 ml). The reaction was terminated by boiling for 5 min. The resultant l-β -EHAsp was converted into l-β-EHAsn in a reaction mixture (10 ml) containing 500 mM HEPES-NaOH Buffer (pH 8.4), 25 mM MgCl_2_, 25 mM ATP, 25 mM NH_4_Cl, 5% (v/v) glycerol, and 4 mg of *E. coli* AsnA at 37°C for 24 h. Then, the pH value was adjusted to 4.0 with acetic acid and the supernatant was applied to a Dowex 50W-X8 column (H^+^ form, 100–200 mesh, 1.3 × 3 cm) previously equilibrated with a 10 mM ammonium acetate buffer (pH 4.5). l-β-EHAsn was eluted with 125 mM ammonium acetate buffer (pH 4.5) and then concentrated under reduced pressure. Purity and concentration of l-β-EHAsn were determined by HPLC as described above.

### Inhibition assay of serine racemase

The initial velocity of l-serine dehydration catalyzed by mouse SR was monitored by a coupled assay with a lactic dehydrogenase (LDH) as described previously [25]. The LDH-coupled assay was carried out in a reaction mixture containing 50 mM HEPES-NaOH Buffer (pH 8), 20 µM PLP, 1 mM MgCl_2_, 1 mM ATP, 0.3 mM NADH, 10 units LDH, 1-10 mM l-Ser, and various concentrations of l-β-EHAsn (0, 50, 100, 200 µM) at 30°C. The reaction was initiated by the addition of mouse SR and NADH consumption was followed at 340 nm. All kinetic parameters were calculated by GraphPad Prism software (GraphPad Software Inc., CA, U.S.A.).

## Results and discussion

### Dsd1p degrades β-hydroxyasparagine in human urine

**Figure 1:**
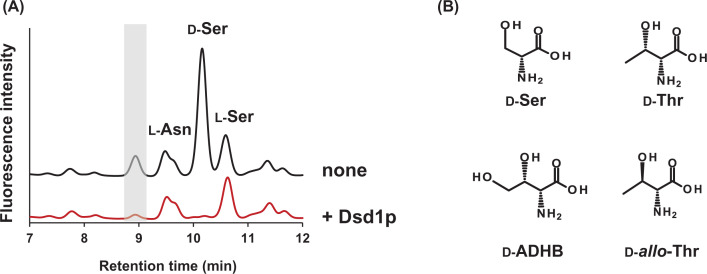
Occurrence of an unknown amino acid in human urine that is degraded by Dsd1p (**A**) Chromatogram of HPLC amino acid analyses of human urine treated with Dsd1p or untreated. Amino acids were derivatized with NAC/OPA and separated using an ODS column by HPLC as described in ‘Materials and methods’ section. (**B**) Structure of known substrates of Dsd1p.

Human urine samples treated with DSD isolated from *S. cerevisiae* (Dsd1p) were analyzed using HPLC. In addition to d-serine, a novel amino acid whose derivative appeared transiently at 8.9 min disappeared by the enzyme treatment (Figure 1A). The detected level of the novel amino acid was comparable with that of proteinogenic l-amino acids in human urine (∼50 μM). Dsd1p degrades d-amino acids that bear a hydroxyl group at C_β_, such as d-Ser, d-Thr, d-*allo*-Thr, and the (*2R, 3S*)-isomer of 2-amino-3,4-dihydroxybutanoic acid (d-ADHB) (Figure 1B). The elution profile of these d-amino acids was different from that of the urinary amino acids. The novel urinary amino acid was further evaluated to identify whether it served as a substrate to d-amino acid transaminase (DAAT) or DAO. Both enzymes showed broad substrate specificity among d-amino acids and could degrade various d-amino acids under appropriate reaction conditions [29,30]. A significant difference was not detected in the concentration of the unknown amino acid after treatment with DAAT or DAO (data not shown). Besides, co-chromatography with a variety of l-amino acids failed to identify the novel amino acid. These results indicated the presence of a novel Dsd1p substrate in human urine.

**Figure 2:**
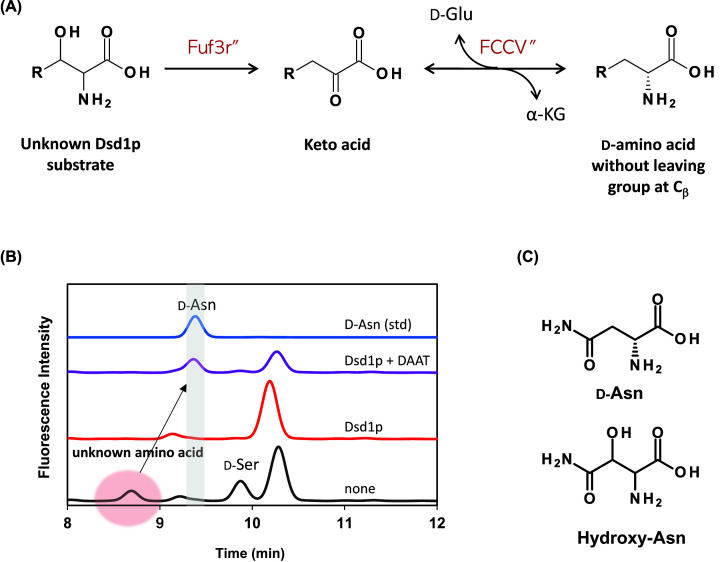
Identification of the urinary Dsd1p substrate as β-hydroxyl asparagine (**A**) Scheme of enzymatic conversion of the Dsd1p substrate into a d-amino acid lacking leaving group at C_β_. Dsd1p probably dehydrates the unknown amino acid to generate corresponding keto acid and ammonia. The keto acid is converted to the corresponding d-amino acid by the action of DAAT. (**B**) Analysis of amino acid generated by the treatment of the Dsd1p and/or DAAT. Urinary amino acids were partially purified and incubated in the absence or presence of Dsd1p and/or DAAT. The amino acids were analyzed by HPLC as described in ‘Materials and methods’ section. A chromatogram of d-Asn standard was also shown. (**C**) Structures of d-Asn and β-hydroxyl asparagine are shown.

Dsd1p catalyzes the α,β-elimination reaction of the substrate amino acid leading to the formation of the corresponding α-keto acid and ammonia (Figure 2A). It was predicted that DAAT could convert the α-keto acid into a d-amino acid lacking the C_β_-cleavage group (Figure 2A). Treatment of the novel urinary amino acid (which was partially purified using anion- and cation-chromatography) with Dsd1p and DAAT in the presence of d-Ala generated a new amino acid whose derivative exhibited a retention time (RT) of 9.3 min. Co-chromatography with 19 kinds of d-amino acids (stereoisomers of proteinogenic amino acids), d-Thr, d-homoserine, d-Orn, d-Ile, or d-aminobutyrate, revealed the detected RT to be consistent with that of d-Asn (Figure 2B).

**Figure 3:**
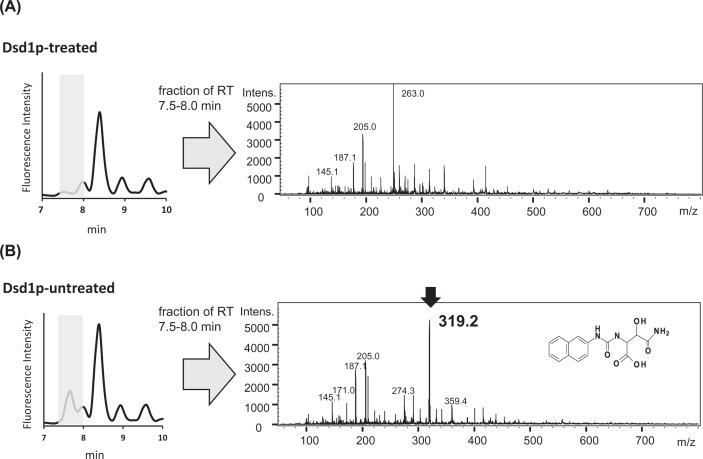
LC-ESI-MS analysis of AQC-derivative of the urinary Dsd1p substrate Partially purified amino acid extract of human urine was incubated in the presence (**A**) or absence (**B**) of Dsd1p. Then amino acids were derivatized with AQC and separated with an ODS column. Eluates from the ODS column (RT 7.5–8.0, shade) were collected and subjected to ESI-MS analysis. The estimated structure of the AQC derivative of β-hydroxyl asparagine is represented.

To determine the molecular mass of the urinary Dsd1p substrate, the amino acids were derivatized with 6-aminoquinolyl-carbamyl (AQC) and subjected to mass spectrometry. This was followed by reverse-phase chromatography, and the fraction comprising the AQC-derivatized target amino acid (RT 7.5–8.0 min) was analyzed by mass spectrometry using an electrospray ionization source. As a control, a similar experiment was conducted using the Dsd1p-treated sample. Comparison of the MS spectra indicated that the *m/z* ratio of the target amino acid derivative was 319.2 (Figure 3). Typically, AQC-amino acids generate a daughter ion with an *m/z* ratio of 171, corresponding to the AQC moiety of the derivative. Results of the present study revealed a characteristic daughter ion corresponding to the AQC moiety (*m/z* = 171) by fragmentation of the ion (MS/MS analysis), confirming that this molecule was indeed an AQC-amino acid. Consequently, the data indicated the estimated molecular mass of the amino acid to be 148.1, which was consistent with the value of β-hydroxyasparagine (β-OH-Asn). Taken together, these results strongly supported that the urinary amino acid decomposed by Dsd1p was β-OH-Asn (Figure 2C).

### Stereochemistry of β-hydroxyasparagine in human urine

The stereochemistry of β-OH-Asn in human urine was unknown. β-OH-Asn has two chiral centers at C_α_ and C_β_ and thus has four stereoisomers: (*2S, 2S*), (*2S, 2R*), (*2R, 2S*), and (*2R, 2R*). Co-chromatography with each of the isomers would have been a simple method of identifying the stereochemistry of the urinary Dsd1p substrate. However, none of the stereoisomers of β-OH-Asn is commercially available.

**Figure 4:**
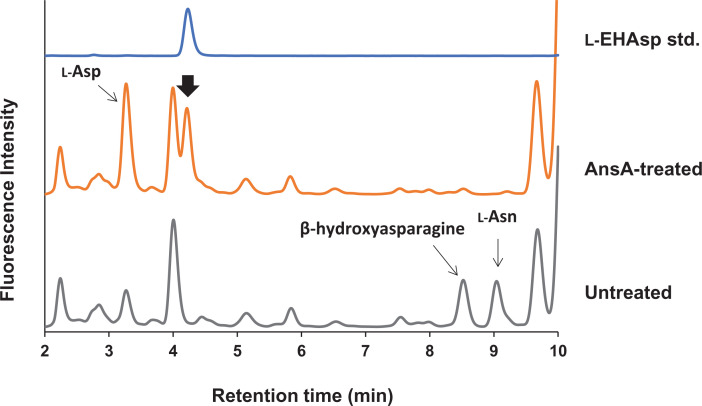
Determination of stereochemistry of urinary β-hydroxy-asparagine The urinary amino acids were incubated in the presence or absence of the *E. coli* asparaginase 1 (AsnA) and analyzed by HPLC. The peaks corresponding to l-Asn (RT 9 min) and β-hydroxyl asparagine (RT 8.5 min) disappeared, and new peaks corresponding to l-Asp (RT 3.3 min) and l-β-hydroxy aspartate (l-β-EHAsp, RT 4.5 min) were generated.

Urinary β-OH-Asn could be converted into the corresponding isomer of β-hydroxy aspartate (β-OH-Asp) using the *E. coli* asparaginase AsnA, which catalyzes the hydrolysis of l-Asn to l-Asp. The new amino acid formed was identified as the (*2S, 3S*)-isomer of the β-OH-Asp (l-*erythro*-β-hydroxyaspartate) by comparing the RT of the four isomers of β-hydroxyaspartate (β-OH-Asp) standards (Figure 4). Consequently, the urinary Dsd1p substrate was identified as the (*2S, 3S*)-isomer of β-OH-Asn (l-β-EHAsn). The occurrence of the (*2S, 3S*)-isomer of β-OH-Asn in human urine has been reported previously [31,32], further supporting our conclusion.

**Figure 5:**
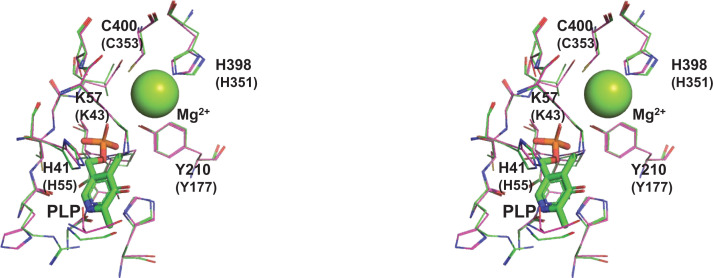
Putative active site structure of Dsd1p The three-dimensional structure of Dsd1p was modeled using the SWISS-MODEL program at the Expasy server (https://swissmodel.expasy.org/). Reported structures of d-*threo*-3-hydroxyaspartate dehydratase from *Delftia* sp. HT23 (d-THA-DH, PDB ID: 3WQC) was used as modeling templates. A stereogram of superposition of putative active site of Dsd1p and the d-THA-DH. Sidechains for the residues of Dsd1p and the d-THA-DH (in parentheses) are labeled. Coloring is magenta for Dsd1p and green for d-THA-DH (3WQC). Visualization and analysis of the structures were carried out using the Pymol software.

As described previously, known substrates of Dsd1p are generally d-amino acids that bear a hydroxyl group at C_β_. The reaction mechanism of Dsd1p associated with the α, β-elimination reaction of l-β-EHAsn was of particular interest. Maeda et al. reported that d-*threo*-3-hydroxyaspartate dehydratase obtained from *Delftia* sp. HT23 (*Delftia* d-THA-DH) exhibited ∼25% sequence identity to Dsd1p degrades both d-*threo*-β*-*hydroxyaspartate [(*2R, 3R*)-isomer] as well as l-β-EHAsp [33]. The crystal structure of *Delftia* d-THA-DH complexed with substrate l-β-EHAsp or inhibitor d-β-EHAsp exhibits a close/distal location of C_β_-OH of l-β-EHAsp/d-β-EHAsp to the catalytically essential divalent metal ion in the active site [34]. This observation suggested that the substrate specificity of *Delftia* d-THA-DH was determined based on the orientation of the substrate C_β_-OH at the active site. The 3D-structural model of Dsd1p constructed using *Delftia* d-THA-DH as a template indicated that Dsd1p exhibited nearly similar active site geometry to *Delftia* d-THA-DH, thus explaining the ability of Dsd1p in utilizing l-β-EHAsn as a substrate (Figure 5).

### Distribution and urinary kinetics of l-β-EHAsn

Although the occurrence of l-β-EHAsn in human urine was reported more than 50 years ago, limited information is available in relation to its physiological distribution and functions. The present study examined the *in vivo* occurrence of l-β-EHAsn in the plasma, urine, and various organs/tissues, including kidney, liver, cerebrum, cerebellum, and testis, in a rat model using HPLC with the Dsd1p treatment. l-β-EHAsn was found at a relatively high concentration in urine in rats (∼60 nmol l-β-EHAsn /mg creatinine) and humans. In contrast, the concentration of l-β-EHAsn was below the detection limit (less than 0.1 μM) in the plasma, cerebrum, cerebellum, kidney, liver, and testis. In the present study, the diet fed to the rats was confirmed to contain negligible free l-β-EHAsn, indicating the endogenous origin of this amino acid. In addition, the low and relatively high concentrations of l-β-EHAsn in the plasma and urine, respectively, might suggest inefficient uptake of l-β-EHAsn by the kidney tubules.

**Figure 6:**
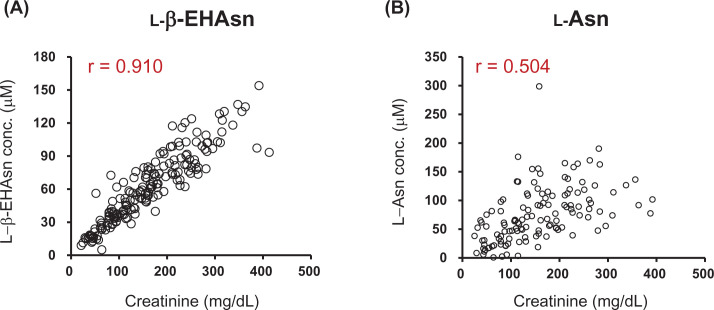
Relationships between urinary concentrations of l-β-EHAsn, l-Asn, and creatinine The first-morning urine and random spot urine at noon and night for 3 consecutive days were collected from young healthy volunteers (*n*=20, age 21–25, male/female = 1/1), and analyzed by HPLC. The concentrations were 34 ± 9 µM. There was a strong correlation between the urinary concentration of l-β-EHAsn and creatinine (r = 0.91) (**A**), and whose values were 4.5 ± 0.5 (nmol l-β-EHAsn/mg creatinine). No correlations between urinary excretion of l-Asn and creatinine were observed (**B**).

Furthermore, the kinetics of l-β-EHAsn in human urine was investigated. The first-morning urine and random spot urine at noon and night were provided by young healthy volunteers (*n*=20, age 21–25, male/female = 1/1) for 3 consecutive days, and subjected to HPLC analyses. All urine samples analyzed had l-β-EHAsn, and the concentration varied significantly among samples. Further analyses revealed a strong correlation between the urinary l-β-EHAsn concentration and creatinine level (r = 0.91), with a median value of 37.9 nmol l-β-EHAsn/mg creatinine (IQR, 33.5–43.7) (Figure 6). The analyses revealed that the kinetics of urinary l-β-EHAsn showed minimal variation among healthy individuals. In contrast, a significant correlation was not observed between the urinary excretion of l-Asn and creatinine (Figure 6).

The origin of urinary l-β-EHAsn in rats and human is currently unknown. *3R*-specific hydroxylation of specific Asn residues in epidermal growth factor (EGF)-like domains in proteins has been identified in mammals [35,36]. This modification is introduced by aspartyl (asparaginyl) β-hydroxylase [37]. Additionally, hydroxylation of an Asn residue in the C-terminal transcriptional activation domain (CAD) of hypoxia-induced factor (HIF) has been reported. This modification prevents CAD from interacting with the p300 transcriptional co-activator [38]. Furthermore, l-β-EHAsn residues have been discovered in a few antifungal peptides, such as theonellamides and xylocandins [39,40]. Proteolysis of these modified proteins/peptides could liberate l-β-EHAsn *in vivo*. Further studies are required to clarify the origin of this amino acid.

### Preparation of (*2S, 3S*)-isomer of l-β-EHAsn

Further study of the novel urinary amino acid necessitated the development of a simple and efficient synthetic method. Limited methods aimed at synthesizing l-β-EHAsn have been reported previously. Sendai et al. synthesized l-β-EHAsn from (-)-*trans*-epoxysuccinic acid, which was obtained by the fermentation of glucose by *Aspergillus fumigatus* [41]. In contrast, Wong et al. obtained β-EHAsn from methyl *p*-methoxycinnamate by complete chemical synthesis in seven steps, with an overall yield of 23% [42].

**Figure 7:**
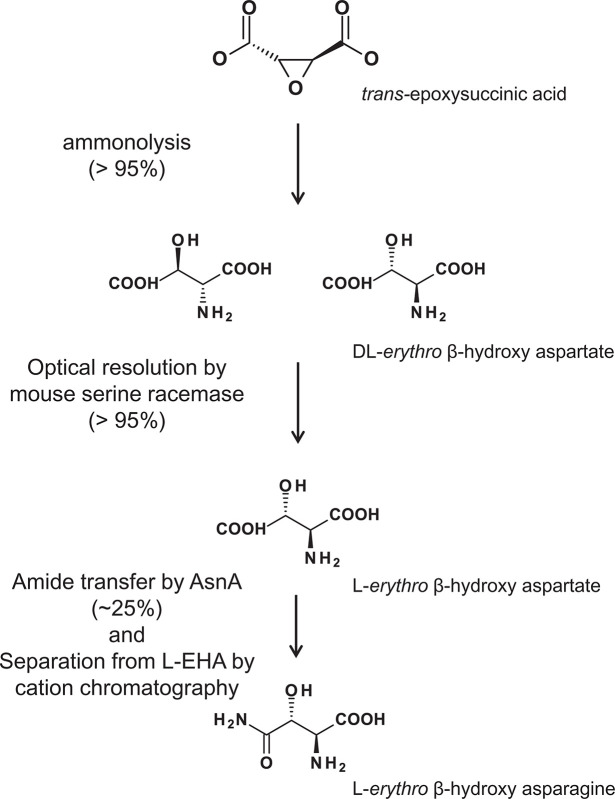
Scheme for preparation of l-β-EHAsn dl-β-EHAsp was obtained by ammonolysis of *trans*-epoxysuccinic acid. d-β-EHAsp was decomposed by mouse SR. l-β-EHAsp was converted into l-β-EHAsn by the action of the AsnA. l-β-EHAsp and l-β-EHAsn can be separated with Dowex 50W-X8 column.

In the present study, we developed a simple three-step synthetic method for the production of l-β-EHAsn coupled with enzymatic resolution and amide transfer (Figure 7). Step 1: A racemic mixture of β-EHAsp (dl-β-EHAsp) was obtained by ammonolysis of (±)-*trans*-epoxysuccinic acid, as described by Jones et al. [43]. Step 2: The racemic mixture was resolved into l-β-EHAsp using mouse SR. Nagano et al. reported an optical resolution of d- and l-β-EHAsp with d-*erythro*-β-hydroxyaspartate dehydratase obtained from *Pseudomonas* sp. N99 [43]. Accordingly, mouse SR exhibiting 35% sequence identity to the *Pseudomonas* enzyme could efficiently eliminate d-β-EHAsp from the racemic mixture. The efficiency of the first two steps was greater than 95%. Step 3: l-β-EHAsp was further converted to l-β-EHAsn by the action of *E. coli* AsnA, resulting in a yield of approximately 25%. The low yield of this step might be attributed to the instability of the AsnA enzyme. Several attempts made to increase the enzyme stability (pH control, changing composition of the reaction mixture, and/or addition of enzyme stabilizers such as glycerol), failed to achieve a significant increase in the productivity of l-β-EHAsn. However, l-β-EHAsn could be separated from l-β-EHAsp by cation chromatography using stepwise elution with ammonium acetate.

### l-β-EHAsn is an inhibitor of serine racemase

**Figure 8:**
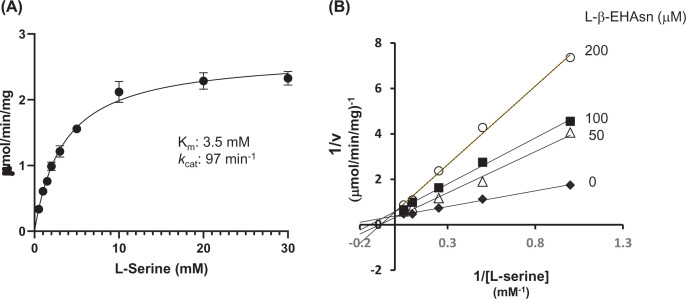
Inhibition of mouse serine racemase by l-β-EHAsn (**A**) Michaelis–Menten plot of the l-Ser β-elimination catalyzed by mouse SR. *K*_m_ and *k*_cat_ values for the l-Ser dehydration were determined to be 3.5 ± 0.4 mM and 97 ± 4 min^−1^, respectively. Data were represented as means ± SD (error bars) of three independent experiments. (**B**) The Lineweaver–Burk plot of the l-Ser β-elimination reaction on inhibitor l-β-EHAsn concentration (0, 50, 100, 200 µM). The l-Ser β-elimination activity was inhibited by l-β-EHAsn with the *K*_i_ value of 40 ± 6 µM, which was estimated by global fitting to a mixed model inhibition equation using GraphPad Prism software α value of 19, R^2^ = 0.98). Note that the data was also well fitted to a competitive inhibition equation (R^2^ = 0.98). Reactions were carried out as described in Materials and Methods. Each data point represents mean of two independent experiments.

The regulation of SR *in vivo*, which is responsible for the production of d-Ser and to a lesser extent d-Asp, continues to be of immense scientific interest. d-Ser serves as a co-agonist of the NMDA receptor, and hypo- or hyperactivation of the NMDA receptor is involved in certain neurological diseases. Since SR is the primary enzyme that supplies endogenous d-Ser, inhibitors that curb d-Ser synthesis could be used for the treatment of diseases caused by the NMDA receptor hyperactivation. SR activity is modulated by energy levels (ATP and NADH), metal ions (Mg^2+^ and Ca^2+^), post-translational modifications (phosphorylation *O*-palmitoylation and *S*-nitrosylation), and protein interactors [44–45]. Stříšovský et al. reported that certain dicarboxylic substrate analogs, such as malonate and l-β-EHAsp, could exhibit a strong competitive inhibitory effect on mouse SR, wherein the latter compound had a *K*_i_ value of 49 µM [46]. In addition, l-Asn and Gly inhibited SR activity with a *Ki* value of ∼2 mM, and the latter is suggested as a physiologically relevant inhibitor [46,47]. The inhibitory effect of l-β-EHAsn on SR was predicted based on the structural similarity of l-β-EHAsn and l-β-EHAsp. To examine this possibility, we prepared recombinant mouse SR and performed inhibition studies based on the l-Ser β-elimination activity at various concentrations of l-Ser and l-β-EHAsn. The steady-state kinetic parameters for β-elimination of l-Ser catalyzed by the mouse SR (3.5 ± 0.4 mM for *K*_m_ and 97 ± 4 min^−1^ for *k*_cat_ values) were nearly identical with those in previously reported data (4.0 mM for *K*_m_ and 81 min^−1^ for *k*_cat_ values, [46]) (Figure 8A). Furthermore, a kinetic study demonstrated mixed-mode inhibition of SR by l-β-EHAsn with a *K*_i_ value of 40 ± 6 µM (Figure 8B).

## Conclusion

The present study demonstrated that l-β-EHAsn, an atypical amino acid abundantly present in mammalian urine, is served as a substrate of Zn^2+^-dependent DSD. Quantitative analyses of the urinary l-β-EHAsn in young healthy volunteers revealed a correlation between urinary l-β-EHAsn concentration and creatinine level. We also described a simple three-step synthetic method of l-β-EHAsn and a strong inhibitory effect of this amino acid on mammalian SR. Elucidation of the precise distribution of this amino acid may shed light on the physiological role of l-β-EHAsn including control of SR reaction.

## Data Availability

The datasets used and/or analyzed during the current study are available from the corresponding author on reasonable request.

## Abbreviations

AsnA: asparagine synthase
AQC: 6-Aminoquinoline *N*-succinimidyl ester
CAD: C-terminal transcriptional activation domain
DAAT: d-amino acid transaminase
DAO: d-amino acid oxidase
DSD: d-serine dehydratase
HPLC: high-performance liquid chromatography
LDH: lactic dehydrogenase
l-β-EHAsn: l-*erythro*-β-hydroxyasparagine
NMDA: *N*-methyl-d-aspartate
RT: retention time
TCA: trichloroacetic acid
β-OH-Asn: β-hydroxyasparagine
β-OH-Asp: β-hydroxyaspartate
